# Severe Suprasystemic Refractory Pulmonary Hypertension in a Neonate with Stüve–Wiedemann Syndrome Associated with Biallelic LIFR Variants: Molecular Insights and a Neonatal Case Report

**DOI:** 10.3390/ijms27177824

**Published:** 2026-08-31

**Authors:** Karolina Chojnacka, Greta Sibrecht, Karolina Gruca-Stryjak, Tomasz Szczapa

**Affiliations:** 1 Neonatal Biophysical Monitoring and Cardiopulmonary Therapies Research Unit, II Department of Neonatology, Poznan University of Medical Sciences, 61-701 Poznan, Poland; greta.sibrecht@gmail.com (G.S.); tszczapa@gmail.com (T.S.); 2 Department of Perinatology, Gynecology and Obstetrics, Poznan University of Medical Sciences Clinical Hospital, 60-535 Poznan, Poland; karolagruca@poczta.onet.pl; 3 Genesis Medical Genetics Center, 60-529 Poznan, Poland; 4 Department of Perinatology, Obstetrics and Gynecology, Polish Mother’s Memorial Hospital Research Institute, 93-338 Łódź, Poland

**Keywords:** Stüve–Wiedemann syndrome, *LIFR* gene, persistent pulmonary hypertension of the newborn, endothelial-to-mesenchymal transition (EndMT), pulmonary vascular remodeling, echocardiography, neonatal intensive care

## Abstract

Stüve–Wiedemann syndrome (SWS) is an ultra-rare autosomal recessive skeletal dysplasia caused by loss-of-function variants in the leukemia inhibitory factor receptor (*LIFR*) gene. While characterized by bone deformities and dysautonomia, severe persistent pulmonary hypertension of the newborn (PPHN) significantly contributes to high early mortality. We report a neonate with genetically confirmed SWS who presented with severe, suprasystemic PPHN refractory to standard pulmonary vasodilators, including inhaled nitric oxide. This case provides a detailed longitudinal hemodynamic characterization of severe suprasystemic PPHN in genetically confirmed SWS, including serial assessment of pulmonary pressures, shunt direction, and right ventricular function during treatment. Rather than identifying PPHN as a novel manifestation of SWS, it extends the phenotypic and hemodynamic characterization of pulmonary vascular involvement in this rare disorder.

## 1. Introduction

Stüve–Wiedemann syndrome (SWS; MIM #211970) is an ultra-rare, autosomal recessive skeletal dysplasia caused by loss-of-function variants in the leukemia inhibitory factor receptor (*LIFR*) gene on chromosome 5p13 [1]. The condition is clinically characterized by a distinct triad: severe bowing of the long bones with joint contractures, profound autonomic dysregulation, and immediate neonatal respiratory distress [1,2]. Impairment of the LIFR-dependent downstream signaling pathways (JAK/STAT3 and PI3K/Akt) disrupts both skeletal morphogenesis and autonomic nervous system development, explaining its complex multisystem phenotype [1].

Although skeletal malformations facilitate early clinical diagnosis, overall prognosis is dictated by early-onset respiratory failure, which accounts for most of the neonatal mortality [2]. Traditionally attributed to restrictive thoracic hypoplasia or central dysautonomia, emerging clinical evidence indicates that severe, concomitant persistent pulmonary hypertension of the newborn (PPHN) significantly contributes to this high mortality. Notably, SWS-associated PPHN is notoriously refractory to standard pulmonary vasodilators, including inhaled nitric oxide (iNO) and phosphodiesterase-5 inhibitors [3]. LIFR deficiency may also affect the pulmonary vasculature through endothelial-to-mesenchymal transition (EndMT) and vascular remodeling, although the relevance of these mechanisms in individual patients is still unclear [3].

Herein, we report the case of a neonate with genetically confirmed Stüve–Wiedemann syndrome complicated by severe, suprasystemic PPHN. We focus on the longitudinal echocardiographic characterization of pulmonary pressures, shunt direction, and ventricular function during treatment, and discuss the diagnostic and therapeutic challenges raised by this course. Because PPHN has previously been reported in SWS, this case is intended to extend the hemodynamic and phenotypic characterization of pulmonary vascular involvement rather than to define a novel manifestation of the syndrome.

## 2. Case Report

A male neonate was delivered by cesarean section to a primigravida mother (G1P1). Gestational age was estimated at 38 weeks based on first-trimester crown–rump length measurement. Cesarean delivery was performed because of prenatally detected skeletal abnormalities. Grade I fetal growth restriction was also noted.

Prenatal ultrasound demonstrated shortening and bowing of the long bones, particularly the femora and humeri, together with thoracic hypoplasia and shortened ribs, resulting in a reduced thoracic circumference. Abnormal positioning and contractures of the hands and feet raised suspicion of arthrogryposis. Bone mineralization was preserved, with no evidence of fractures. The skull, clavicles, and scapulae appeared normal. Fetal growth restriction and oligohydramnios were noted in late pregnancy. Prenatal chromosomal microarray analysis showed no pathogenic copy-number variants. The parents declined prenatal whole-exome sequencing.

**Figure 1:**
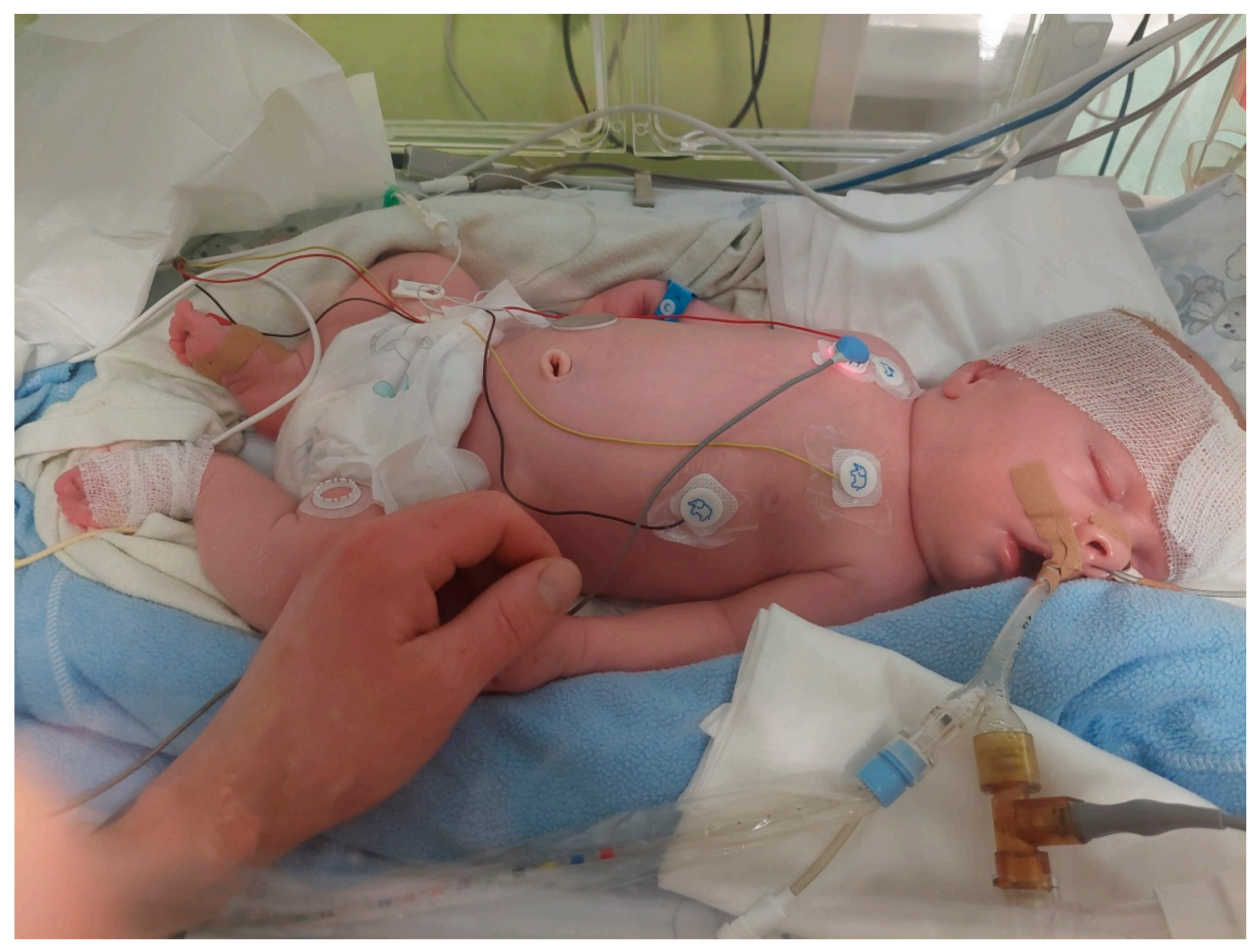
Clinical presentation of the neonate during the acute phase of persistent pulmonary hypertension of the newborn (PPHN). The patient is intubated and managed with pressure-regulated volume control (PRVC) ventilation combined with inhaled nitric oxide (iNO) therapy. Continuous monitoring included electrocardiography (ECG), transcutaneous carbon dioxide (PtcCO_2_) monitoring, cerebral near-infrared spectroscopy (NIRS), and simultaneous pre- and post-ductal pulse oximetry. Central venous access was established via a catheter placed in the right femoral vein.

Apgar scores were 3, 3, 6, and 9 at 1, 3, 5, and 10 min of life, respectively. Umbilical cord blood gas pH values were within the normal range, and the patient was born with a birth weight of 3440 g. Physical examination revealed dysmorphic features, including a hypoplastic chest, short palpebral fissures, hypertelorism, a flat facial profile, and low-set ears. Skeletal radiographs demonstrated abnormal long-bone morphology with widened metaphyses and narrowed diaphyses. Abnormal positioning of the fingers and toes was noted, including adducted thumbs and ulnar deviation of the hallux and toes (Figure 1).

The infant was initiated on non-invasive ventilation (NIV) in the delivery room and transferred to the neonatal intensive care unit (NICU). At 5 h of life, due to worsening respiratory distress and an FiO_2_ requirement of 0.55, he required endotracheal intubation. Over the following hours, oxygen requirements progressively increased, and systemic blood pressure fell. Serial echocardiograms confirmed severe suprasystemic PPHN (detailed parameters in Section 3). The infant was ventilated in neurally adjusted ventilatory assist (NAVA) mode. The following day, FiO_2_ requirements remained at approximately 0.70 on iNO at 20 ppm and attempts to wean iNO were unsuccessful.

On day 3 of life, oxygen requirements increased to FiO_2_ 1.0, with severe respiratory distress requiring high-dose sedation. Oxygen saturation remained around 90% or lower, and catecholamine support was initiated. Saturation subsequently fell to 20%, prompting transition to high-frequency oscillatory ventilation with volume guarantee (HFOV + VG) and escalation of iNO to 25 ppm. Laboratory testing showed elevated inflammatory markers, leading to antibiotic therapy; coagulation abnormalities were treated with fresh frozen plasma, and intravenous pentaglobin was administered. Pneumothorax and severe intracranial hemorrhage were excluded. Despite FiO_2_ 1.0, oxygen saturation remained approximately 40%, with mixed acidosis and an oxygenation index (OI) of 48 sustained for more than 4 h. Pre-ductal peripheral oxygen saturation (SpO_2_) was approximately 50–60% and post-ductal SpO_2_ 60–70%, with a mean arterial pressure of 49 mmHg and preserved urine output. Although the infant met clinical criteria for extracorporeal membrane oxygenation (ECMO), extracorporeal support was not initiated because of extreme clinical instability and the high risk of inter-hospital transport to an ECMO center. Fever up to 39.2 °C and insulin-refractory hyperglycemia were also recorded on day 3.

On day 4, cranial ultrasound demonstrated abnormal cerebral blood flow with a resistive index (RI) of 0.4, consistent with severe hypoxic–ischemic injury. Amplitude-integrated electroencephalography (aEEG) showed a flat tracing approaching the isoelectric line. On day 5, as SpO_2_ normalized, FiO_2_ was gradually reduced to approximately 0.75. Acidosis progressively improved, and catecholamine doses were reduced. The infant remained heavily sedated, with narrow pupils that were non-reactive to light. Occasional extensor limb movements were observed, but there was otherwise no spontaneous motor activity.

In the following days, FiO_2_ was further reduced. An attempt to decrease iNO from 25 to 20 ppm at FiO_2_ 0.40 was unsuccessful, and recurrent fever episodes unresponsive to paracetamol continued to occur. iNO was eventually discontinued on day 8. Nutrition was entirely parenteral throughout admission, except for the first day of life. Pupils regained reactivity to light on day 10, and the remaining catecholamines were gradually weaned from day 11. The infant required albumin infusions and multiple packed red blood cell transfusions during admission. On day 12, rising inflammatory markers prompted an antibiotic change and the addition of fluconazole. On day 13, ventilation was transitioned from HFOV + VG to pressure-regulated volume control (PRVC) mode, and from day 14 the infant was ventilated in NAVA mode with FiO_2_ of 0.21. Brain magnetic resonance imaging (MRI) demonstrated extensive hypoxic–ischemic changes.

A comprehensive evaluation was undertaken to identify potential contributors to persistent pulmonary hypertension. Echocardiography demonstrated a structurally normal heart, excluding congenital heart disease. Chest imaging showed thoracic hypoplasia and shortened ribs but no evidence of pulmonary hypoplasia or diaphragmatic abnormality, such as hernia or eventration. There was no history of meconium-stained amniotic fluid and no radiographic evidence of meconium aspiration syndrome. Infection was considered because inflammatory markers were elevated, although blood cultures remained negative throughout the clinical course. Coagulation studies revealed a transiently prolonged INR of 2.1 on day of life 2, which normalized thereafter, with no clinical or imaging findings suggestive of thromboembolic disease. Metabolic testing did not reveal evidence of a metabolic or mitochondrial disorder. Overall, these findings support a multifactorial interpretation of PPHN in this patient; although a mechanistic link between LIFR dysfunction and pulmonary vascular alterations remains biologically plausible, this single-case observation cannot establish a definitive causal relationship.

A summary and timeline of the iNO and treatment course, including the vasoactive and pulmonary vasodilator agents used, are presented in Table 1.

In the subsequent days, sedation was gradually reduced and the infant was extubated to NIV-NAVA, then progressed to nCPAP and finally HFNC. Antibiotics were discontinued on day 23 following normalization of inflammatory markers. On day 27, antibiotics were restarted due to a rise in procalcitonin; central line culture grew *Enterobacter hormaechei* and antibiotic therapy was adjusted according to sensitivity results. Throughout the admission, the infant experienced recurrent episodes of elevated body temperature, typically up to 38 °C and occasionally reaching 41.5 °C, accompanied by tachycardia up to 280 bpm without ECG evidence of supraventricular tachycardia. Management included metamizole and physical cooling measures. Episodic agitation accompanied by high fever and tachycardia required treatment with phenobarbital, and chlorpromazine was also administered following neurological consultation. On day 70 of life, clinical seizures were observed and confirmed on aEEG recording, requiring treatment with phenobarbital and levetiracetam. Intensive rehabilitation was required throughout the entire hospital admission.

**Figure 2:**
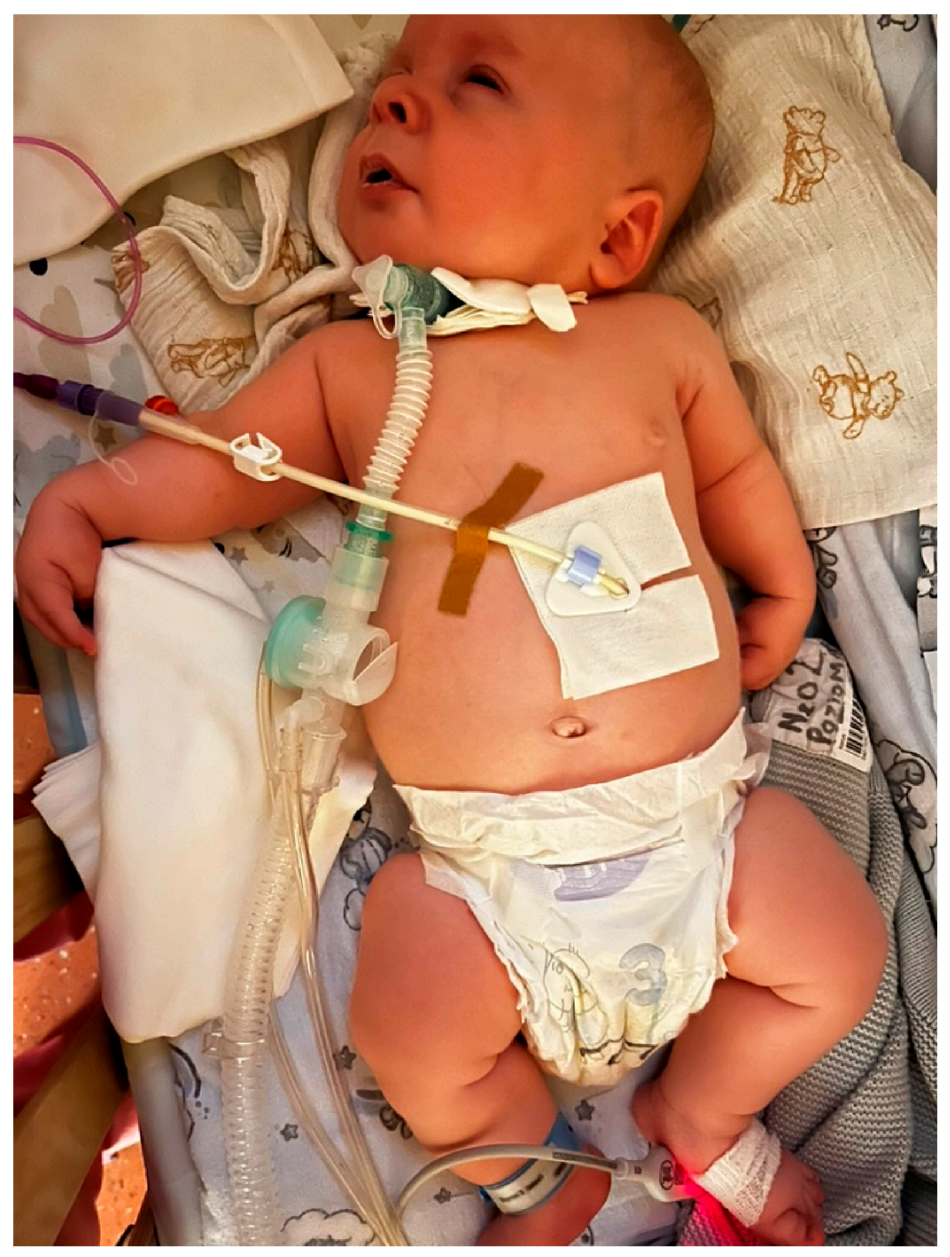
Clinical features of the patient at three months of age after stabilization. The photograph shows midface hypoplasia with a flat facial profile, hypertelorism, short palpebral fissures, micrognathia, low-set dysplastic ears, and a narrow hypoplastic thorax. Shortening of the upper limbs is also visible. Distal limb abnormalities include adducted thumbs and marked deviation of the great toes and digits. The patient had previously undergone tracheostomy and percutaneous endoscopic gastrostomy placement as part of supportive management.

Due to feeding difficulties with the absence of an effective sucking reflex, a percutaneous endoscopic gastrostomy (PEG) tube was placed on day 56 of life, and a tracheostomy was performed on day 65 (Figure 2).

Whole-exome sequencing was performed with analysis of protein-coding exons, exon–intron boundaries, selected non-coding regions, and exon-level copy-number variants. The median sequencing depth was 149×, and 99.82% of the target region was covered at ≥20×. Two pathogenic nonsense variants in LIFR were identified: NM_002310.6:c.2434C>T, p.(Arg812*) and NM_002310.6:c.2074C>T, p.(Arg692*). Both variants were confirmed by Sanger sequencing. Parental testing showed that each parent carried one of the variants, confirming that they were in trans. This finding established the molecular diagnosis of Stüve–Wiedemann syndrome. The family was offered genetic counseling regarding autosomal recessive inheritance, recurrence risk, and options for prenatal or preimplantation genetic testing.

## 3. Echocardiographic Findings and PPHN Diagnosis

**Figure 3:**
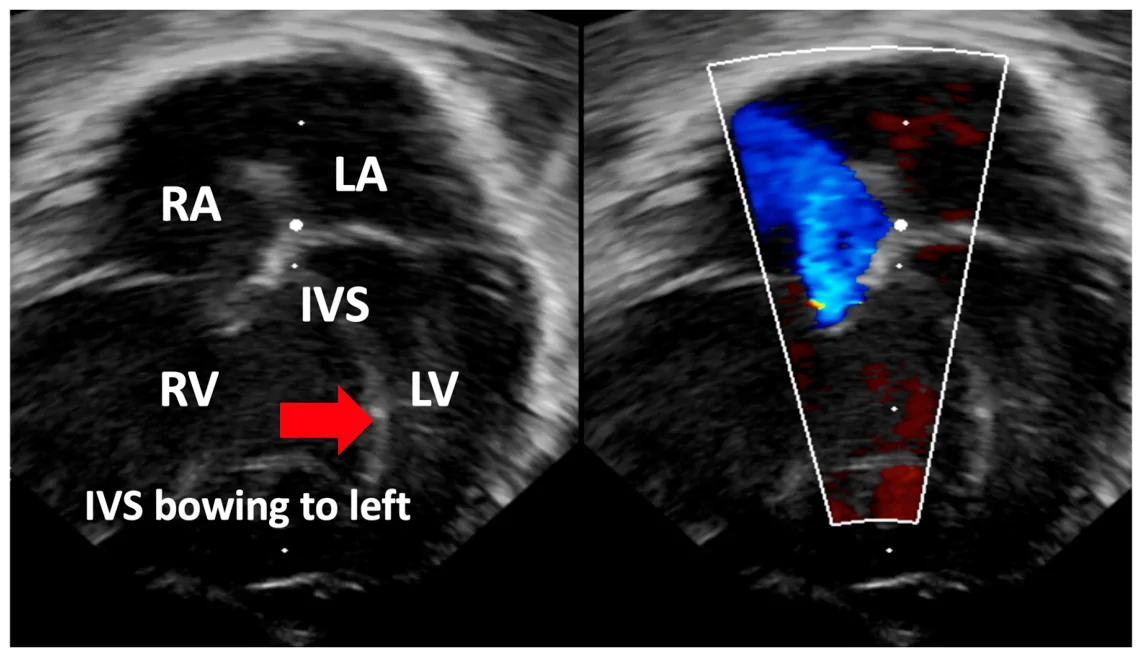
Transthoracic echocardiogram demonstrating severe right ventricular pressure overload. Parasternal short-axis view showing marked right ventricular (RV) dilation and free-wall hypertrophy. Severe elevation of pulmonary arterial pressure results in systemic or suprasystemic RV pressures, causing systolic and diastolic flattening with paradoxical leftward bowing of the interventricular septum (IVS) (arrow), which compresses the left ventricle (LV) into a characteristic D-shaped configuration. Representative image for illustrative purposes. Reprinted with permission from Chojnacka et al., Biomedicines 2025 [4]. Abbreviations: IVS, interventricular septum; LA, left atrium; LV, left ventricle; RA, right atrium; RV, right ventricle.

**Figure 4:**
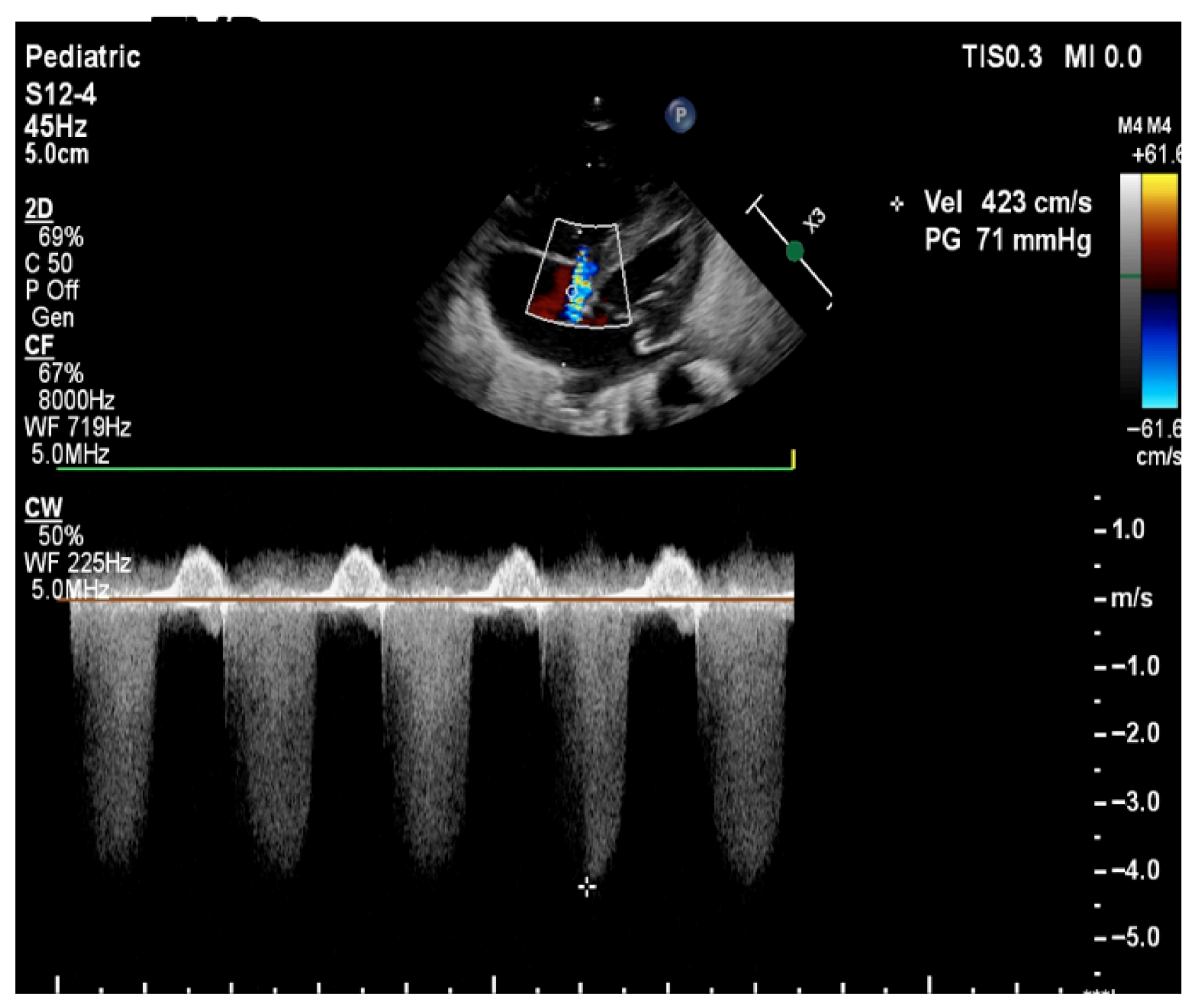
Continuous-wave Doppler quantitative estimation of systolic pulmonary artery pressure (sPAP). Transthoracic echocardiography in the apical four-chamber view demonstrates a high-velocity tricuspid regurgitation (TR) jet. Continuous-wave Doppler interrogation across the tricuspid valve reveals a high-velocity regurgitant flow profile corresponding to an elevated peak pressure gradient between the right ventricle and right atrium, illustrating severe pulmonary hypertension. Representative image for illustrative purposes. Reprinted with permission from Chojnacka et al., Biomedicines 2025 [4].

**Figure 5:**
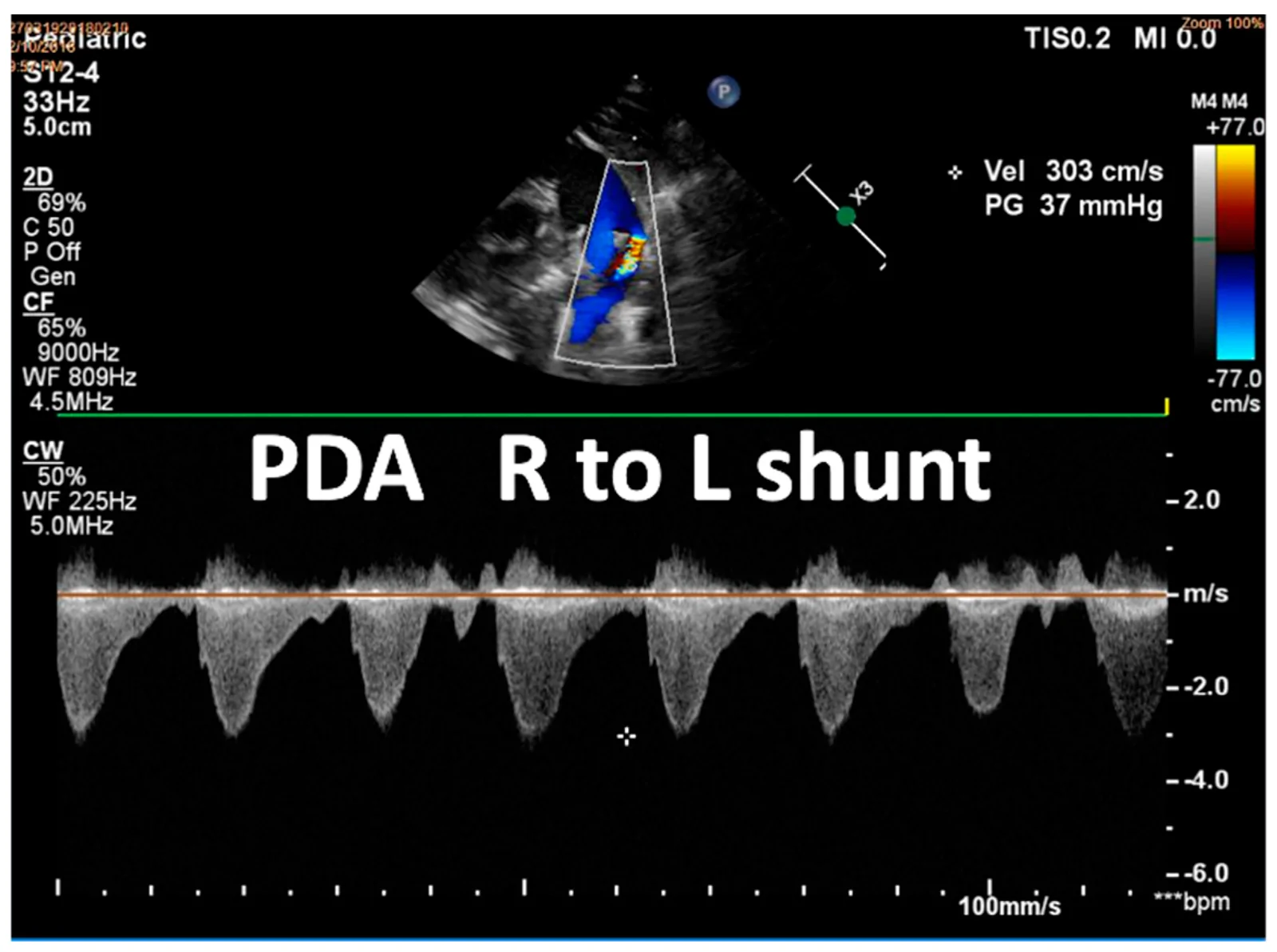
Color and spectral Doppler assessment of patent ductus arteriosus (PDA) hemodynamics. High-resolution color Doppler imaging demonstrates a patent ductus arteriosus with a continuous, unrestrictive right-to-left (R–L) shunt. The right-to-left directionality confirms that pulmonary vascular resistance exceeds systemic vascular resistance and illustrates profound post-ductal hypoxemia. Representative image for illustrative purposes. Reprinted with permission from Chojnacka et al., Biomedicines 2025 [4]. Abbreviations: PDA, patent ductus arteriosus; R–L, right-to-left shunt.

**Figure 6:**
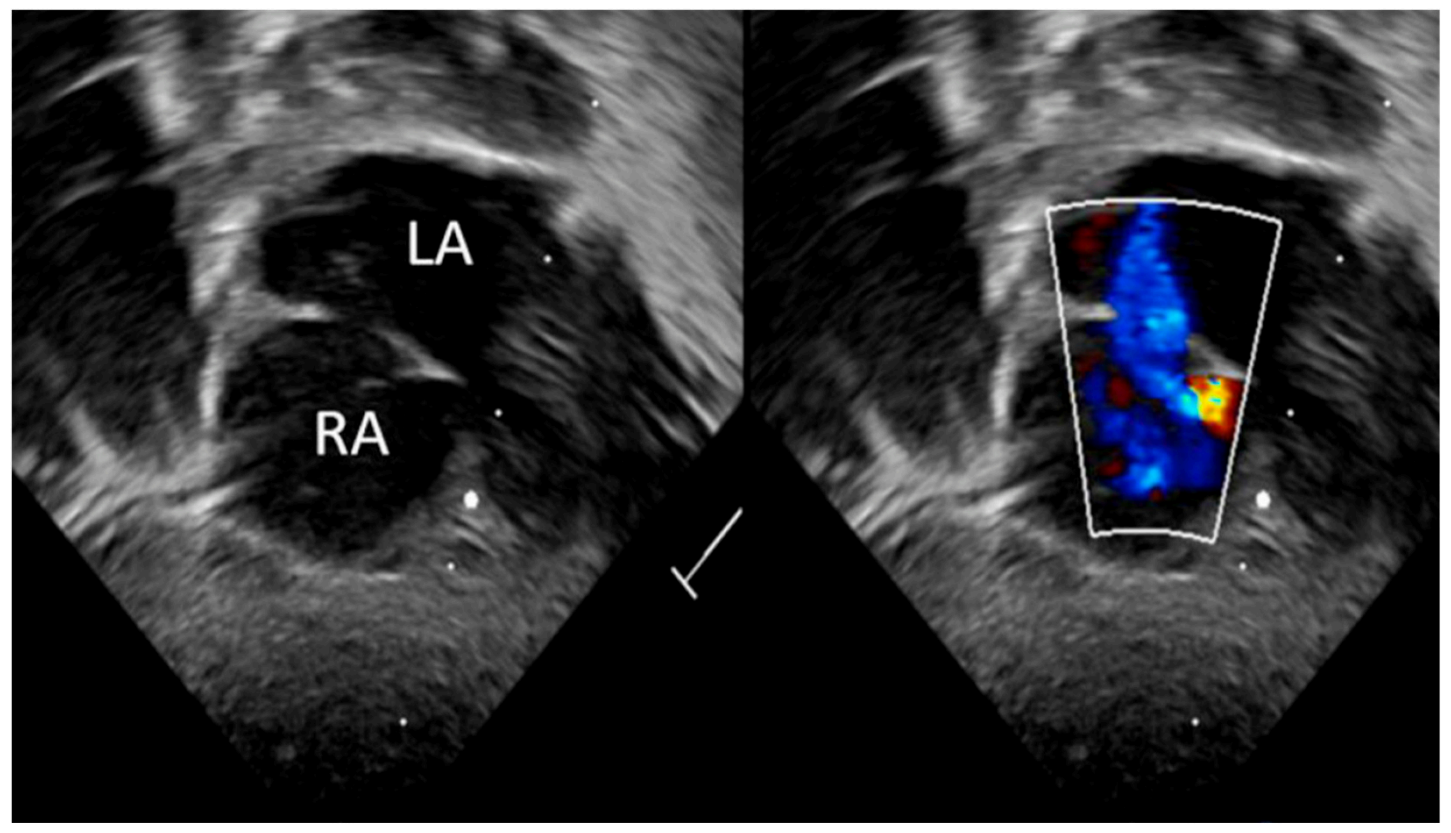
Interatrial shunt directionality at the patent foramen ovale (PFO). Color Doppler interrogation of the interatrial septum in the subcostal view demonstrates bidirectional, predominantly right-to-left (R–L) flow across the patent foramen ovale. Elevated right atrial pressure, secondary to elevated right ventricular end-diastolic pressure and severe PPHN, drives deoxygenated systemic venous blood directly into the left atrium. Representative image for illustrative purposes. Reprinted with permission from Chojnacka et al., Biomedicines 2025 [4]. Abbreviations: LA, left atrium; PFO, patent foramen ovale; PPHN, persistent pulmonary hypertension of the newborn; R–L, right-to-left shunt; RV, right ventricle. The blue color in the Doppler image indicates the direction of shunt flow R-L.

**Figure 7:**
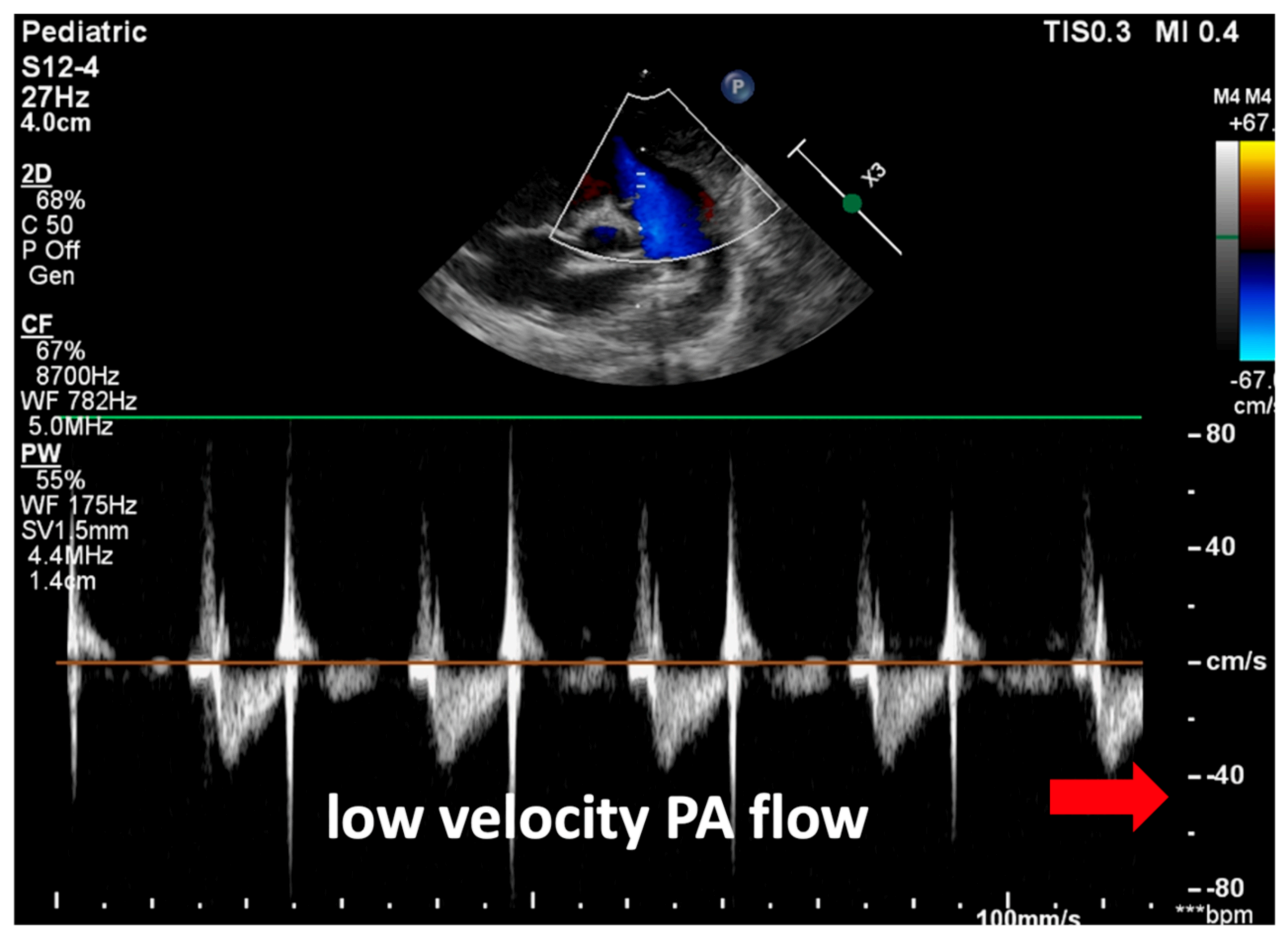
Pulsed-wave Doppler profile of the right ventricular outflow tract (RVOT). Doppler interrogation of pulmonary arterial flow demonstrates a characteristic low-velocity, high-resistance spectral profile (arrow). Note the severe attenuation of peak flow velocity and the presence of mid-systolic notching, a feature of significantly increased pulmonary vascular impedance. Representative image for illustrative purposes. Reprinted with permission from Chojnacka et al., Biomedicines 2025 [4].

**Figure 8:**
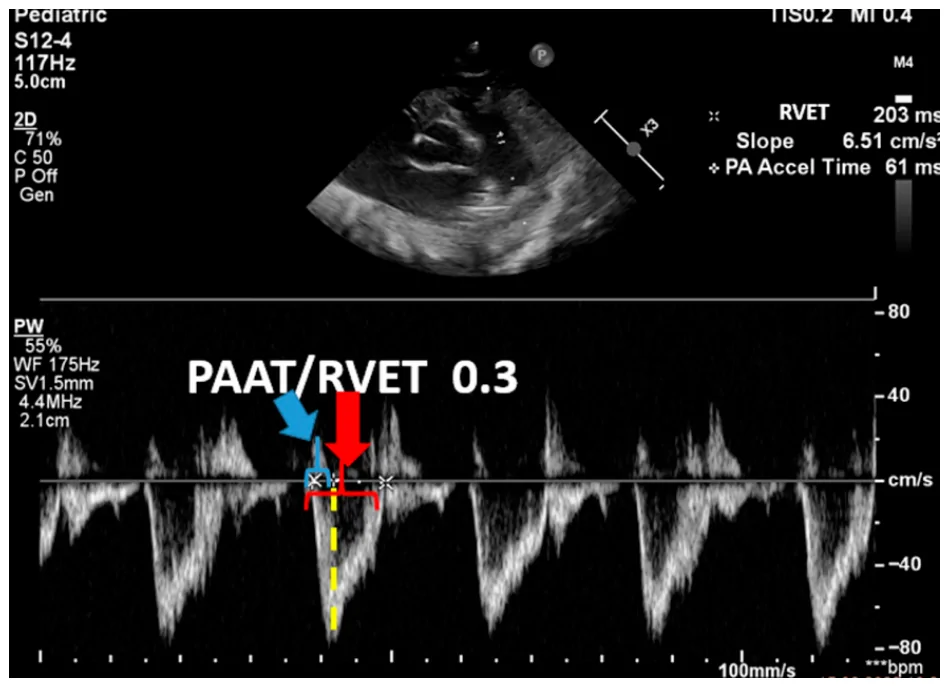
Quantitative pulmonary arterial Doppler analysis. Quantitative evaluation of the pulmonary artery flow signal demonstrates a markedly shortened pulmonary artery acceleration time (PAAT), measured between the white dashed lines, and an abnormally low PAAT/right ventricular ejection time ratio (PAAT/RVET; approximately 0.3), indicated by the yellow bracket. Representative image for illustrative purposes. Reprinted with permission from Chojnacka et al., Biomedicines 2025 [4]. Abbreviations: PAAT –pulmonary artery acceleration time (blue arrow); RVET, right ventricular ejection time (red arrow).

Echocardiography (ECHO) was central to the diagnosis and longitudinal hemodynamic monitoring of PPHN in this patient. Serial bedside assessments were used to track pulmonary vascular resistance, shunt direction, ventricular function, and response to vasoactive and vasodilator therapy. The diagnostic approach used in our department is summarized below. Figure 3, Figure 4, Figure 5, Figure 6, Figure 7 and Figure 8 are representative echocardiographic images from our previous work [4], included to illustrate the classical features of severe neonatal PPHN physiology observed in the present patient.

1. Exclusion of Structural Anomalies: Initial comprehensive echocardiographic mapping confirmed normal segmental anatomy and explicitly ruled out congenital heart defects, including Total Anomalous Pulmonary Venous Connection (TAPVC). This confirmed that the neonate’s profound hypoxemia was functional rather than structural in origin.
2. Chamber Geometry and Visual Function: On visual inspection (“eyeballing”), severe right ventricular (RV) enlargement and marked hypertrophy of the RV free wall were observed, indicating marked acute pressure and volume overload (Figure 3).
3. Interventricular Septal Kinetics: Evaluation of the interventricular septum (IVS) revealed extreme flattening during ventricular systole. Reflecting the severity of the pulmonary overpressure, the left ventricle (LV) exhibited a distinct crescent-shaped configuration, demonstrating that the right ventricular and pulmonary artery pressures markedly exceeded systemic arterial pressures (Figure 3).
4. Pulmonary Artery Pressure Quantification: A prominent tricuspid regurgitation (TR) jet was identified. Quantitative estimation using the modified Bernoulli equation ($\Delta P=4V_{max}^{2}$) with an assumed right atrial pressure (RAP) of 5 mmHg yielded an estimated Systolic Pulmonary Artery Pressure (sPAP) indicative of severe, supra-systemic pulmonary hypertension. Suprasystemic pulmonary pressure was defined as an estimated sPAP exceeding the concurrent systemic systolic blood pressure (Figure 4).
5. Shunt Hemodynamics and Directionality: Color and continuous-wave Doppler interrogation of the transitional pathways revealed critical right-to-left shunting through both the patent foramen ovale (PFO) and the patent ductus arteriosus (PDA). This right-to-left directionality at both levels confirmed the diagnosis of very severe PPHN and accounted for the refractory central cyanosis (Figure 5 and Figure 6).
6. Quantitative Right Ventricular Indices: Advanced functional assessment documented secondary RV myocardial impairment. The Tricuspid Annular Plane Systolic Excursion (TAPSE) was markedly reduced, and Tissue Doppler Imaging (TDI) demonstrated an elevated RV Tei index. Furthermore, a markedly decreased RV fractional area change (FAC %) was observed.
7. Left Ventricular Performance: The left ventricle showed a secondary reduction in performance, with decreased fractional shortening (FS) and ejection fraction (EF), alongside an altered LV Tei index. This deterioration was attributed to the severe septal shift and impaired left ventricular filling (interventricular dependence).
8. Advanced Hemodynamic Metrics: Detailed analysis of the pulmonary vasculature showed a profoundly shortened pulmonary arterial acceleration time (PAAT) and an abnormally low PAAT/RV ejection time ratio. Additionally, speckle tracking and strain rate imaging identified a critical reduction in global and regional right ventricular longitudinal strain, providing a baseline for monitoring response to aggressive pulmonary vasodilator therapy (Figure 7 and Figure 8).

These findings are consistent with markedly elevated pulmonary vascular resistance and increased pulmonary vascular impedance in the setting of severe PPHN. Although these indices support the presence of severe pulmonary vascular dysfunction, they do not distinguish between potentially reversible vasoconstriction and fixed structural pulmonary vascular remodeling.

The detailed progression of serial echocardiographic parameters, pulmonary pressures, and clinical hemodynamics across the hospital course is summarized in Table 2.

Despite the successful resolution of the pulmonary hypertensive crisis and the discontinuation of respiratory and vasoactive support, the infant experienced severe hypoxic–ischemic brain injury with subsequent seizures, required long-term tracheostomy and gastrostomy, and was ultimately discharged under palliative home care in the third month of life.

## 4. Discussion

In our patient, SWS was complicated by severe suprasystemic PPHN, profound hypoxemia, and marked right ventricular pressure overload. The initial response to pulmonary vasodilator therapy was limited. Although the oxygenation index reached 48 and persistent right-to-left shunting prompted consideration of ECMO, the infant gradually stabilized with intensive respiratory and hemodynamic support. Serial echocardiography was used to follow pulmonary pressure, shunt direction, and ventricular function during the acute phase.

Persistent pulmonary hypertension of the newborn (PPHN) is a clinical syndrome characterized by the failure of the normal circulatory transition at birth, leading to sustained elevation of pulmonary vascular resistance and right-to-left shunting of blood through the foramen ovale and ductus arteriosus. This pathophysiology results in severe and often labile hypoxemia. The etiology of PPHN is classified into three primary patterns: maladaptation, where structurally normal vessels fail to dilate; maldevelopment, characterized by medial hypertrophy of the pulmonary arterioles; and underdevelopment, involving a reduced number of pulmonary vessels [4,5,6,7,8]. Diagnosis of PPHN requires a high index of clinical suspicion and the exclusion of cyanotic congenital heart disease, with echocardiography serving as the gold standard for assessment [9,10,11]. Key diagnostic findings include evidence of right-to-left or bidirectional shunting and signs of right ventricular pressure overload, such as flattening or paradoxical bowing of the interventricular septum toward the left ventricle [9,10,11]. The severity of respiratory failure is often assessed using the oxygenation index (OI), while indices such as PAAT and eccentricity index provide quantitative evaluation [9,10,11].

Pulmonary hypertension is not unique to the present case and has previously been reported in SWS. However, the published cases differ substantially in genotype, severity of pulmonary vascular involvement, treatment response, and outcome. To contextualize the present findings, Table 3 summarizes selected molecularly or clinically characterized SWS cases that are relevant to pulmonary vascular involvement and genotype–phenotype interpretation.

This comparison indicates that pulmonary hypertension, including severe neonatal PPHN, has been reported previously in SWS and therefore should not be considered a novel manifestation of the disorder. Rather, the distinctive contribution of the present case is the detailed longitudinal hemodynamic characterization of severe suprasystemic PPHN in a neonate carrying the specific compound-heterozygous LIFR c.2434C>T, p.(Arg812*) and c.2074C>T, p.(Arg692*) genotype. Serial echocardiography documented suprasystemic pulmonary pressures, right-to-left ductal and interatrial shunting, marked right ventricular pressure overload, secondary ventricular dysfunction, and their evolution during treatment.

The available cases also demonstrate substantial phenotypic variability among patients with LIFR variants. Notably, p.(Arg692*) has also been reported in compound heterozygosity in a patient with a substantially milder, later-onset phenotype without reported neonatal pulmonary hypertension [1]. Thus, although the specific p.(Arg812*)/p.(Arg692*) combination has not, to our knowledge, been reported previously, the severity of pulmonary vascular disease cannot currently be attributed to either individual variant or to this specific genotype. Additional cases are required to determine whether particular LIFR genotypes influence the risk or severity of pulmonary vascular involvement.

Management of PPHN traditionally follows a pragmatic approach aimed at optimizing systemic oxygen delivery while minimizing lung injury [16,17,18,19,20]. Therapeutic strategies prioritize gentle ventilation, while inhaled nitric oxide (iNO) serves as the first-line selective pulmonary vasodilator [16,17,18,19,20]. In non-responders, a multimodal approach incorporating phosphodiesterase inhibitors like sildenafil, milrinone, and hemodynamic support with inotropes and vasopressors is required to ensure systemic blood pressure exceeds pulmonary pressure [16,17,18,19,20].

In our patient, echocardiography showed marked right ventricular pressure overload. Right ventricular dilation and hypertrophy, paradoxical septal bowing, and right-to-left shunting through the ductus arteriosus and foramen ovale indicated pulmonary pressures at or above the systemic level. Reduced TAPSE, fractional area change, and right ventricular longitudinal strain were consistent with secondary right ventricular dysfunction. Serial measurements were used to guide treatment and follow hemodynamic improvement.

In neonates with SWS, pulmonary hypertension may be severe and may respond incompletely to conventional pulmonary vasodilator therapy. This limited response may reflect a greater contribution of prenatal pulmonary vascular remodeling than in PPHN caused predominantly by reversible vasoconstriction.

Biallelic loss-of-function variants in LIFR impair cytokine signaling through pathways that include JAK/STAT3 [3,21,22,23]. This signaling defect may interfere with normal pulmonary vascular development and contribute to abnormal vascular remodeling. Proposed mechanisms include endothelial-to-mesenchymal transition, medial hypertrophy, extension of smooth muscle into normally non-muscularized distal arterioles, and reduced growth of the peripheral pulmonary vascular bed [21,22,23]. Together, these changes could reduce the total cross-sectional area and compliance of the pulmonary circulation, limiting the normal postnatal fall in pulmonary vascular resistance.

The mechanisms responsible for PPHN in SWS remain incompletely understood. Endothelial-to-mesenchymal transition is a recognized contributor to vascular remodeling in pulmonary hypertension, but its specific role in LIFR deficiency has not been established in affected neonates and was not directly assessed in our patient. The clinical course may therefore reflect a combination of prenatal structural remodeling and a reversible component of pulmonary vasoconstriction. A predominantly structural pulmonary vascular lesion could limit the response to vasodilator therapy because pharmacological relaxation cannot fully reverse medial hypertrophy, reduced distal vascular growth, or advanced remodeling. However, the relative contribution of fixed remodeling and reversible vasoconstriction is likely to vary between patients. Treatment should therefore be guided by serial assessment of pulmonary pressure, shunt direction, ventricular function, and systemic perfusion rather than by an assumption of universal drug resistance.

While LIFR-related pulmonary vascular remodeling represents a plausible contributor to the PPHN observed in this infant, the underlying phenotype might be multifactorial. This infant also presented with thoracic hypoplasia and shortened ribs, which may have contributed to impaired pulmonary mechanics, as well as severe respiratory failure immediately after birth, both of which can independently promote hypoxic pulmonary vasoconstriction and secondary PPHN. In addition, the clinical course was complicated by infection, which is also a recognized trigger of PPHN through mechanisms including endothelial dysfunction, cytokine-mediated vasoconstriction, and impaired nitric oxide bioavailability. In the present infant, inflammatory markers (IL-6 and CRP) were negative initially, but CRP began to rise toward the end of day of life (DOL) 1, reaching 63.7 mg/L on DOL 2 and peaking at 83 mg/L on DOL 12; procalcitonin peaked earlier, on DOL 3, at 74.3 ng/mL; and IL-6 peaked at 1011 pg/mL on DOL 12, coinciding with the CRP peak, despite antibiotic therapy. These findings raise the possibility that infection overlapped with and may have aggravated the pulmonary vascular phenotype. Yet, we did not observe a clear correlation between the severity or trend of these inflammatory markers and the severity of PPHN in this infant, arguing against infection as the dominant driver. Taken together, we favor a multifactorial mechanism in which LIFR-mediated pulmonary vascular remodeling, thoracic hypoplasia with impaired pulmonary mechanics, perinatal respiratory failure, and superimposed inflammatory/infectious insults may have contributed to the severity and persistence of pulmonary hypertension in this patient, rather than attributing the entire pulmonary vascular phenotype to LIFR-mediated remodeling alone.

The gradual clinical improvement suggests that pulmonary vascular dysfunction in our patient was at least partly reversible. Although shortened PAAT and reduced PAAT/RVET ratios indicate markedly increased pulmonary vascular load, these functional Doppler metrics cannot, by themselves, establish that pulmonary vascular disease is fixed or anatomically irreversible. This distinction is underscored by our patient’s subsequent clinical improvement, including the gradual normalization of oxygen requirements, successful discontinuation of iNO, reversal of shunt direction, normalization of interventricular septal kinetics, and complete withdrawal of vasoactive support. Thus, the clinical course is most compatible with a significant, dynamic, and reversible component of pulmonary vasoconstriction, potentially superimposed on underlying developmental or structural vascular abnormalities associated with LIFR deficiency.

These observations raise the possibility that LIFR deficiency may contribute to pulmonary vascular remodeling and thereby modify the severity or reversibility of PPHN, but this mechanism remains hypothetical and cannot be established from a single case. In severe neonatal PPHN, alternative therapies remain limited. Prostacyclin-pathway agents such as iloprost or treprostinil may be considered in selected cases, whereas endothelin receptor antagonists such as bosentan are often impractical during the acute neonatal phase because of their slower onset and oral route of administration. From a molecular perspective, restoration of LIFR/STAT3 signaling is an experimental concept rather than a clinically available therapy, and it is unknown whether prenatal intervention would be required to prevent structural vascular changes. ECMO remains a rescue option for refractory hypoxemic respiratory failure and right ventricular compromise when transfer and initiation are feasible.

Although the described infant met physiological criteria for ECMO consideration (persisting with an oxygenation index >40 for over four hours), actual transfer and initiation of extracorporeal support were considered unsafe due to profound hemodynamic instability and acute critical deterioration. This case illustrates how limited access to on-site ECMO impacts treatment options in critically ill neonates and emphasizes the importance of early consultation with an ECMO center when severe PPHN is suspected.

No disease-modifying treatment for LIFR deficiency is currently available. Experimental readthrough of premature termination codons using gentamicin has resulted in partial restoration of functional LIFR activity in cultured fibroblasts, but this approach has not been evaluated clinically in patients with SWS [24]. It also remains uncertain whether restoration of LIFR signaling could modify the full phenotype, when such treatment would need to be initiated, whether pulmonary vascular remodeling is already established prenatally in all affected patients, or whether LIFR-targeted therapy would be safe. These experimental findings therefore cannot be considered evidence that molecular treatment would prevent or reverse PPHN. Current management remains supportive and should be individualized according to respiratory status, pulmonary hemodynamics, ventricular function, feeding safety, and autonomic complications.

The clinical and molecular understanding of Stüve–Wiedemann syndrome has changed substantially over the last two decades, particularly since Dagoneau et al. showed that null variants in the *LIFR* gene abolish JAK/STAT3 pathway signaling [1]. Current data from the systematic review by Warnier et al. indicate that mortality is highest during the first two years of life, with a reported mortality rate of 42% before age two and 10% thereafter; pulmonary arterial hypertension was associated with a poor prognosis, with a mortality rate of 63% [25]. Respiratory failure and dysautonomic complications, including hyperthermic episodes, remain major contributors to early morbidity and mortality [25].

Recurrent hyperthermia was a major feature of the clinical course. Body temperature reached 41.5 °C and was accompanied by marked sinus tachycardia and agitation, with limited response to standard antipyretic treatment. These episodes were compatible with dysautonomia in SWS and required physical cooling and symptomatic treatment. Their interpretation was complicated by concurrent inflammatory episodes and bloodstream infection, which made it difficult to distinguish autonomic hyperthermia from infection-related fever.

Pulmonary hypertension has been reported in a minority of patients with SWS but appears to be associated with poor outcomes. In the systematic review by Warnier et al., pulmonary arterial hypertension was identified as a poor prognostic factor, with a reported mortality rate of 63% [25]. Severe neonatal PPHN and pulmonary hypertensive crises have also been described in individual cases [14,15,22,26,27].

For patients surviving beyond age two, mortality appears substantially lower [13,14,25,26,27,28,29]. In this later period, the clinical picture becomes a profoundly interdisciplinary problem dominated by progressive orthopedic challenges, including spinal deformities, severe osteoporosis, and recurrent fractures, alongside autonomic deterioration such as corneal anesthesia, reduced pain perception, persistent swallowing difficulties, and motor delay [25,26]. Stomatological challenges such as tongue-biting injuries may occur due to lack of pain sensation [25,26]. Notably, despite severe physical and autonomic disabilities, cognitive development is often preserved, which is a crucial consideration for long-term multidisciplinary care and clinical decision-making [25,26].

## 5. Genetic Findings

Whole-exome sequencing identified two heterozygous pathogenic nonsense variants in *LIFR*. Variant nomenclature was based on the reference transcript NM_002310.6, and sequence reads were aligned to the GRCh37/hg19 human reference genome. The variants were NC_000005.9:g.38485984G>A; NM_002310.6:c.2434C>T, p.(Arg812*) and NC_000005.9:g.38490385G>A; NM_002310.6:c.2074C>T, p.(Arg692*). To our knowledge, the specific c.2434C>T/c.2074C>T combination has not been reported previously.

Whole-exome sequencing was performed using hybridization-based target capture followed by Illumina sequencing-by-synthesis with paired-end reads (2 × 150 bp). Reads were aligned using Burrows–Wheeler Aligner maximal exact matches (BWA-MEM), and variant calling was performed using Genome Analysis Toolkit (GATK)-based algorithms. The median sequencing depth was 149×, and 99.82% of the target region was covered at ≥20×. Variant annotation included population and disease-specific databases, including Genome Aggregation Database (gnomAD), ClinVar, and Human Gene Mutation Database (HGMD).

The c.2434C>T, p.(Arg812*) variant introduces a premature termination codon in exon 17 of 20 and was present in 1/251,394 alleles in gnomAD. This variant has previously been reported in the homozygous state in a patient with Stüve–Wiedemann syndrome [13]. The c.2074C>T, p.(Arg692*) variant introduces a premature termination codon in exon 15 of 20 and was present in 12/278,548 alleles in gnomAD v2. It has previously been reported in affected individuals with Stüve–Wiedemann syndrome, including in compound heterozygosity in a patient with a milder phenotype [1]. ClinVar entries for both variants are listed separately in the references [30,31].

Both variants are predicted to result in loss of normal LIFR function through protein truncation and/or nonsense-mediated mRNA decay, consistent with the established loss-of-function mechanism of LIFR-related Stüve–Wiedemann syndrome. MutationTaster predicted both variants to be disease-causing, whereas SIFT and PolyPhen were not applicable to these nonsense variants.

Both variants were classified as pathogenic according to American College of Medical Genetics and Genomics/Association for Molecular Pathology (ACMG/AMP) criteria (PVS1_VeryStrong, PM3) [29]. PVS1_VeryStrong was applied because both nonsense variants introduce premature termination codons predicted to result in loss of function in a gene for which loss of function is an established disease mechanism. Both variants were confirmed by Sanger sequencing. Parental testing demonstrated that each variant was inherited from a different parent, confirming their occurrence in trans and establishing compound heterozygosity, supporting application of PM3 in this autosomal recessive disorder.

No clear genotype–phenotype correlation has been established in SWS, and patients carrying truncating LIFR variants may differ substantially in disease severity and survival [1,25,28].

Pulmonary vascular involvement may therefore also depend on other genetic, developmental, or environmental factors.

Identification of the familial variants enabled accurate genetic counseling. For each future pregnancy, the recurrence risk is 25%, with a 50% chance of an unaffected carrier and a 25% chance of a child inheriting neither familial variant. Targeted prenatal diagnosis or preimplantation genetic testing may be considered.

## 6. Conclusions

Severe PPHN is an uncommon but previously reported manifestation of Stüve–Wiedemann syndrome. The present case contributes a detailed longitudinal hemodynamic characterization of severe suprasystemic PPHN, including serial changes in pulmonary pressure, shunt direction, and right ventricular function during treatment, together with molecular characterization of a previously unreported compound-heterozygous LIFR genotype. The observed clinical and hemodynamic improvement indicates that severe pulmonary hypertensive crises in SWS may include a dynamic and clinically reversible vasoconstrictive component, potentially superimposed on developmental or structural pulmonary vascular abnormalities. Although the specific p.(Arg812*)/p.(Arg692*) variant combination has not, to our knowledge, been reported previously, the available evidence does not support a direct relationship between this genotype and the severity of pulmonary vascular disease. This case therefore extends the phenotypic and hemodynamic characterization of pulmonary vascular involvement in SWS rather than establishing PPHN as a novel manifestation of the syndrome.

## 7. Limitations

Interpretation of the treatment response is constrained by the limited evidence available for SWS. Published data are largely confined to case reports and small observational series, and no clinical trials have specifically assessed pulmonary vasodilators or molecularly targeted therapies in this population. Treatment decisions must therefore be individualized and are generally based on established principles of neonatal PPHN management.

In addition, invasive hemodynamic assessment and histopathological examination of the pulmonary vasculature were not performed. Consequently, our diagnosis and ongoing hemodynamic monitoring relied entirely on non-invasive serial echocardiography, and we were unable to determine the relative contribution of reversible vasoconstriction and fixed vascular remodeling to the observed pulmonary hypertension. Alternatively, severe pulmonary hypertension may represent a secondary complication of acute critical illness, prolonged hypoxia, or systemic stress rather than a direct, primary manifestation of LIFR pathway disruption.

## Figures and Tables

**Table 1:** Longitudinal treatment timeline during the hospital course.

DOL	iNO (ppm)	Noradrenaline (µg/kg/Min)	Arginine Vasopressin (mU/kg/Min)	Adrenaline (µg/kg/Min)	Milrinone (µg/kg/Min)
1	20	-	-	-	-
2	20–25	0.05	0.2	0.5	0.75
3	25	-	0.5	0.5	0.75
4	25	-	0.5	0.5	0.75
5	25	-	0.5	0.4	0.75
6	20–10	-	0.4	0.37	0.69
7	10–5	-	0.3	0.35	0.66
8	5–0	-	0.2	0.34	0.64
9	-	-	-	0.34	0.5
10	-	-	-	0.34	-
11	-	-	-	-	-

Note: DOL, day of life; iNO, inhaled nitric oxide. Recommended medication dosage in neonates: Noradrenaline: 0.05–1.0 µg/kg/min; Adrenaline: 0.05–1.0 µg/kg/min; Vasopressin: 0.2–2 mU/kg/min; Milrinone: 0.2–1.0 µg/kg/min.

**Table 2:** Longitudinal serial echocardiographic and clinical evaluations during the hospital course.

DOL	Interatrial Shunt (PFO/FoA)	Ductus Arteriosus (DA/PDA)	Tricuspid Regurgitation (Vmax)	Estimated Hemodynamics and Functional Metrics sPAP, TAPSE, PAAT, PAAT/RVET, RV FAC, RV Strain, LV FS, Systemic sBP	Septal Kinetics & Chamber Morphology	Clinical Echocardiographic & Hemodynamic Summary
1	(L–R)	Patent DA, (L-R)	Minimal TR	Not quantified TAPSE 10 mm PAAT/RVET 0.35 Systemic sBP 43 mmHg	Normal cardiac structures	Normal cardiac morphology; left-to-right physiological shunts.
2 *(early)*	Bidirectional	Patent DA, (R–L)	Grade III TR, Vmax 3.0 m/s	sPAP 41 mmHg TAPSE 6 mm RV FAC 22% RV strain −11% PAAT 40 ms PAAT/RVET 0.25 LV FS 23% Systemic sBP 40 mmHg	Asymmetric chambers (RV > LV) RA enlargement, IVS bowed toward LV	Features of acute PPHN onset. FiO_2_ 1.0
2 *(post-iNO)*	Bidirectional	Patent DA, Bidirectional, predominantly (R–L)	Grade III TR, Vmax 3.4 m/s	sPAP 51 mmHg TAPSE 6 mm RV FAC 23% RV Tei Index 0.58 PAAT 38 ms PAAT/RVET 0.20 LV FS 22% Systemic sBP 45 mmHg	Asymmetric chambers (RV > LV), RA enlargement, IVS bowed toward LV	Moderate-to-severe PPHN following iNO initiation (20 ppm). FiO_2_ 1.0
3	Bidirectional, predominantly (R–L)	Patent DA, Bidirectional, predominantly (R–L)	Grade III TR, Vmax 3.8 m/s	sPAP 63 mmHg TAPSE 6 mm, RV FAC 22% RV strain −10% PAAT 35 ms PAAT/RVET 0.20 LV FS 23% Systemic sBP 50 mmHg	Marked RV overload (RV > LV), RA enlargement, severe IVS flattening/bowing into LV	Severe Suprasystemic PPHN (OI 48); iNO 25 ppm. FiO_2_ 1.0, low SpO_2_
4	(R–L)	Patent DA, (R–L)	Grade III TR, Vmax 3.8 m/s	sPAP 63 mmHg TAPSE 7 mm, RV FAC 25% PAAT 35 ms PAAT/RVET 0.20 LV FS 25% Systemic sBP 50 mmHg	Persistent severe RV pressure overload, IVS flattening	Persistent Suprasystemic PPHN; cranial US showing severe hypoxic–ischemic flow (RI 0.4).
5	(R–L)	Patent DA, (R–L)	Regress of severe peak gradient	sPAP 45 mmHg TAPSE 8 mm PAAT 40 ms PAAT/RVET 0.25 LV FS 28% Systemic sBP 45 mmHg	Improving septal bowing	SpO_2_ normalizing, FiO_2_ weaned to 0.75, mixed acidosis resolving.
8	(L–R)	Closure/Minimal shunt	Minimal TR	sPAP 35 mmHg TAPSE 10 mm PAAT 58 ms PAAT/RVET 0.35 LV FS 32% Systemic sBP 42 mmHg	Septal position normalizing	iNO successfully discontinued after earlier unsuccessful weaning attempts.
14	(L–R)	Closed/Silent	Minimal TR	Near-normal pulmonary pressures TAPSE 11 mm PAAT/RVET 0.38 Systemic sBP 45 mmHg	Normal septal kinetics	NAVA mode ventilation, FiO_2_ 0.21

Abbreviations: DA, ductus arteriosus; DOL, day of life; FAC, fractional area change; FiO_2_, fraction of inspired oxygen; FoA, foramen ovale; iNO, inhaled nitric oxide; IVS, interventricular septum; L–R, left-to-right shunt; LV, left ventricle; LV FS, left ventricular fractional shortening; NAVA, neurally adjusted ventilatory assist; OI, oxygenation index; PAAT, pulmonary artery acceleration time; PAAT/RVET, pulmonary artery acceleration time/right ventricular ejection time ratio; PDA, patent ductus arteriosus; PFO, patent foramen ovale; PPHN, persistent pulmonary hypertension of the newborn; R–L, right-to-left shunt; RA, right atrium; RI, resistive index; RV, right ventricle; RV FAC, right ventricular fractional area change; RVET, right ventricular ejection time; sBP, systolic blood pressure; sPAP, systolic pulmonary artery pressure; SpO_2_, peripheral oxygen saturation; TAPSE, tricuspid annular plane systolic excursion; TR, tricuspid regurgitation; US, ultrasonography; Vmax, peak velocity. Note: Neonatal reference ranges for functional parameters: TAPSE ~8–10 mm; PAAT/RVET ratio > 0.31; RV FAC > 30–35%; RV Tei index < 0.40; LV FS ~28–40%.

**Table 3:** Selected previously reported LIFR-related Stüve–Wiedemann syndrome cases relevant to genotype–phenotype and pulmonary vascular involvement.

Study	*LIFR* Variant(s); Inheritance	Age/Presentation	Skeletal Phenotype	Dysautonomia	Respiratory Manifestations	PH/Cardiac Findings	Treatment	Outcome
Raas-Rothschild et al., 2003 [12]	Genotype NR; report preceded identification of *LIFR* as the causative gene	Neonatal	Typical SWS skeletal phenotype	Hyperthermic episodes	Severe neonatal respiratory compromise	Severe PH; pulmonary arterial wall abnormalities reported	Ventilatory and supportive treatment	Early fatal course reported in an affected neonate
Dagoneau et al., 2004 [13]	Homozygous *LIFR* c.2434C>T, p.(Arg812Ter); AR	Neonatal/infantile	Classical SWS skeletal phenotype	NR for genotype-specific case	Genotype-specific respiratory phenotype NR	Genotype-specific PH/cardiac findings NR	NR	Genotype-specific outcome insufficiently reported
Jin et al., 2024 [14]	Homozygous *LIFR* c.756dup, p.(Lys253Ter); AR	Term neonate; early deterioration	Short/bowed extremities; camptodactyly; metaphyseal abnormalities	Recurrent hyperthermia	Severe respiratory failure; invasive ventilation	Severe PPHN; RV dilatation; D-sign; PH crisis	iNO; inhaled/IV iloprost; vasoactive support; hydrocortisone; alprostadil; later sildenafil	Survived; extubated DOL 14; PH not evident at 11 months
Bhalla et al., 2024 [15]	Homozygous *LIFR* c.1646delG, p.(Gly549GlufsTer4); AR	Term neonate	Long-bone bowing; metaphyseal widening; osteopenia	Hyperthermia/dysautonomia	Early severe respiratory distress; pneumothorax; prolonged respiratory support	Neonatal PPHN	HFOV; vasoactive and corticosteroid therapy; supportive care	Survived neonatal period; alive at reported long-term follow-up
Melnik et al., 2024 [1]	Compound heterozygous *LIFR* c.2074C>T, p.(Arg692Ter) + c.3252del, p.(Trp1085GlyfsTer31); in trans	Mild phenotype from birth; diagnosis in adolescence	Camptodactyly; metaphyseal/orthopedic abnormalities; mild early skeletal phenotype	Later heat intolerance; abnormal sweating	No severe neonatal respiratory phenotype reported	PH not reported	Orthopedic/supportive management	Alive at 15 years
Present case	Compound heterozygous *LIFR* NM_002310.6:c.2434C>T, p.(Arg812*) + c.2074C>T, p.(Arg692*); in trans	Term male neonate	Prenatal long-bone shortening/bowing; thoracic hypoplasia; contractures; widened metaphyses	Hyperthermia to 41.5 °C; tachycardia; feeding/autonomic dysfunction	Severe respiratory failure; prolonged mechanical ventilation	Severe suprasystemic PPHN; R–L PDA/PFO shunting; RV dilatation/hypertrophy; secondary ventricular dysfunction	iNO up to 25 ppm; HFOV + VG; vasoactive support; intensive respiratory therapy	Survived acute PH crisis; tracheostomy/PEG; discharged home in third month

Abbreviations: AR, autosomal recessive; DOL, day of life; HFOV, high-frequency oscillatory ventilation; iNO, inhaled nitric oxide; NR, not reported; PDA, patent ductus arteriosus; PFO, patent foramen ovale; PH, pulmonary hypertension; PPHN, persistent pulmonary hypertension of the newborn; RV, right ventricle; SWS, Stüve–Wiedemann syndrome; VG, volume guarantee. Table note: Data are presented only when they could be attributed to the respective reported case or genotype. The report by Raas-Rothschild et al. preceded identification of *LIFR* as the causative gene of SWS; therefore, no molecular genotype is assigned retrospectively [12]. For the molecular series reported by Dagoneau et al., genotype-specific clinical features are reported only where individual attribution was possible [13].

## Data Availability

The original contributions presented in this study are included in the article. Further inquiries can be directed to the corresponding author.

## References

[1] Melnik E., Sharova M., Kenis V., Morgul A., Zabnenkova V., Markova T. (2024). Case Report: New phenotype of late-onset Stüve–Wiedemann syndrome due to a C-terminal variant in the LIFR gene. Front. Pediatr..

[2] Jung C., Dagoneau N., Baujat G., Le Merrer M., David A., Di Rocco M., Hamel B., Mégarbané A., Superti-Furga A., Unger S. (2010). Stüve-Wiedemann syndrome: Long-term follow-up and genetic heterogeneity. Clin. Genet..

[3] Mikelonis D., Jorcyk C.L., Tawara K., Oxford J.T. (2014). Stüve-Wiedemann syndrome: LIFR and associated cytokines in clinical course and etiology. Orphanet J. Rare Dis..

[4] Chojnacka K., Singh Y., Gahlaut S., Blaz W., Jerzak A., Szczapa T. (2025). Persistent pulmonary hypertension of the newborn: A pragmatic review of pathophysiology, diagnosis, and advances in management. Biomedicines.

[5] Jain A., McNamara P.J. (2015). Persistent pulmonary hypertension of the newborn: Advances in diagnosis and treatment. Semin. Fetal Neonatal Med..

[6] Sankaran D., Lakshminrusimha S. (2022). Pulmonary hypertension in the newborn-etiology and pathogenesis. Semin. Fetal Neonatal Med..

[7] Singh Y., Lakshminrusimha S. (2021). Pathophysiology and management of persistent pulmonary hypertension of the newborn. Clin. Perinatol..

[8] de Boode W.P., Singh Y., Molnar Z., Schubert U., Savoia M., Sehgal A., Levy P.T., McNamara P.J., El-Khuffash A. (2018). Application of neonatologist performed echocardiography in the assessment and management of persistent pulmonary hypertension of the newborn. Pediatr. Res..

[9] Kovacs G., Bartolome S., Denton C.P., Gatzoulis M.A., Gu S., Khanna D., Hassoun P.M., Lau E.M. (2024). Definition, classification and diagnosis of pulmonary hypertension. Eur. Respir. J..

[10] Lakshminrusimha S., Abman S.H. (2024). Oxygen targets in neonatal pulmonary hypertension: Individualized, “precision-medicine” approach. Clin. Perinatol..

[11] Bhattacharya S., Sen S., Levy P.T., Rios D.R. (2019). Comprehensive evaluation of right heart performance and pulmonary hemodynamics in neonatal pulmonary hypertension: Evaluation of cardiopulmonary performance in neonatal pulmonary hypertension. Curr. Treat. Options Cardiovasc. Med..

[12] Raas-Rothschild A., Ergaz-Schaltiel Z., Bar-Ziv J., Rein A.J. (2003). Cardiovascular abnormalities associated with the Stuve-Wiedemann syndrome. Am. J. Med. Genet A.

[13] Dagoneau N., Scheffer D., Huber C., Al-Gazali L.I., Di Rocco M., Godard A., Martinovic J., Raas-Rothschild A., Sigaudy S., Unger S. (2004). Null leukemia inhibitory factor receptor (*LIFR*) mutations in Stüve-Wiedemann/Schwartz-Jampel type 2 syndrome. Am. J. Hum. Genet..

[14] Hamasharef K.H., Najmadden Z.B., Akhailany R., Rashid M.J., Hamakarim K. (2026). Novel Genetic Findings in Stuve‐Wiedemann Syndrome: A Case Report and Review of Literature. Clin. Case Rep..

[15] Bhalla D., Sati S., Basel D., Karody V. (2024). A novel termination site in a case of Stüve–Wiedemann syndrome: Case report and review of literature. Front. Pediatr..

[16] Ruoss J.L., Rios D.R., Levy P.T. (2020). Updates on management for acute and chronic phenotypes of neonatal pulmonary hypertension. Clin. Perinatol..

[17] Verma S., Lumba R., Kazmi S.H., Vaz M.J., Prakash S.S., Bailey S.M., Mally P.V., Randis T.M. (2022). Effects of inhaled iloprost for the management of persistent pulmonary hypertension of the newborn. Am. J. Perinatol..

[18] Janjindamai W., Thatrimontrichai A., Maneenil G., Chanvitan P., Dissaneevate S. (2013). Effectiveness and safety of intravenous iloprost for severe persistent pulmonary hypertension of the newborn. Indian Pediatr..

[19] McNamara P.J., Shivananda S.P., Sahni M., Freeman D., Taddio A. (2013). Pharmacology of milrinone in neonates with persistent pulmonary hypertension of the newborn and suboptimal response to inhaled nitric oxide. Pediatr. Crit. Care Med..

[20] Hansmann G., Koestenberger M., Alastalo T.P., Apitz C., Austin E.D., Bonnet D., Budts W., D’Alto M., Gatzoulis M.A., Hasan B.S. (2019). 2019 updated consensus statement on the diagnosis and treatment of pediatric pulmonary hypertension: The European Pediatric Pulmonary Vascular Disease Network (EPPVDN), endorsed by AEPC, ESPR and ISHLT. J. Heart Lung Transplant..

[21] Lateef O.M., Foote C., Power G., Manrique-Acevedo C., Padilla J., Martinez-Lemus L.A. (2024). LIM kinases in cardiovascular health and disease. Front. Physiol..

[22] Jin J., Rothämel P., Büchel J., Kammer B., Brunet T., Pattathu J., Flemmer A.W., Nussbaum C., Schroepf S. (2024). Case Report: Stüve–Wiedemann syndrome—a rare cause of persistent pulmonary hypertension of the newborn. Front. Pediatr..

[23] Paulin R., Meloche J., Bonnet S. (2012). STAT3 signaling in pulmonary arterial hypertension. JAK-STAT.

[24] Bellais S., Le Goff C., Dagoneau N., Munnich A., Cormier-Daire V. (2010). In vitro readthrough of termination codons by gentamycin in the Stüve-Wiedemann syndrome. Eur. J. Hum. Genet..

[25] Warnier H., Barrea C., Bethlen S., Boon R., Bouniol A., Charni S., Debray F.G., Desir J., Dideberg V., Guissard G. (2022). Clinical overview and outcome of the Stüve-Wiedemann syndrome: A systematic review. Orphanet J. Rare Dis..

[26] Kelly C., Morand M., Ray J.W. (2026). Adult Case of Stüve–Wiedemann Syndrome. Am. J. Med. Genet. Part A.

[27] Ferrari N., Botti A., Moreschi C., Montalenti M., Nobilia M. (2023). Stüve–Wiedemann syndrome in a child: A case report. MedNEXT J. Med. Health Sci..

[28] Bertola D.R., Honjo R.S., Baratela W.A. (2016). Stüve-Wiedemann Syndrome: Update on Clinical and Genetic Aspects. Mol. Syndromol..

[29] Richards S., Aziz N., Bale S., Bick D., Das S., Gastier-Foster J., Grody W.W., Hegde M., Lyon E., Spector E. (2015). Standards and guidelines for the interpretation of sequence variants: A joint consensus recommendation of the American College of Medical Genetics and Genomics and the Association for Molecular Pathology. Genet. Med..

[30] National Center for Biotechnology Information ClinVar; VCV000944487.8, NM_002310.6(LIFR):c.2434C>T (p.Arg812Ter). https://www.ncbi.nlm.nih.gov/clinvar/variation/944487/.

[31] National Center for Biotechnology Information ClinVar; RCV001051726.4, NM_002310.6(LIFR):c.2074C>T (p.Arg692Ter). https://www.ncbi.nlm.nih.gov/clinvar/RCV001051726/.

